# Complete genome sequencing and construction of full-length infectious cDNA clone of *papaya ringspot virus*-HYD isolate and its efficient *in planta* expression

**DOI:** 10.3389/fmicb.2023.1310236

**Published:** 2023-11-30

**Authors:** Prodosh Gupta, Pavani L. C. Parupudi, Laha Supriya, Harshal Srivastava, Gudipalli Padmaja, Kodetham Gopinath

**Affiliations:** Department of Plant Sciences, University of Hyderabad, Hyderabad, India

**Keywords:** papaya ringspot virus, infectious cDNA, double promoter, ribozyme, PR genes, transcription factors

## Abstract

*Papaya ringspot virus* (PRSV) is a devastating *Potyvirus* that causes papaya ringspot disease in *Carica papaya* plantations globally. In this study, the complete genome sequence of a PRSV isolate from Shankarpalli, Telangana, India, was reported and designated as PRSV-HYD (KP743981.1). The genome is a single-stranded positive-sense RNA comprising 10,341 nucleotides. Phylogenetic analysis revealed that PRSV-HYD is closely related to PRSV Pune (Aundh) isolate with 92 and 95% nucleotide and amino acid sequence identity, respectively. To develop infectious cDNA (icDNA), the complete nucleotide sequence of PRSV-HYD was cloned between the right and left borders in the binary vector pCB301 using *Bgl*II and *Xma*I restriction sites. *Cauliflower mosaic virus* (CaMV) double promoter (35S) was fused at the 5′-end and *Avocado sunblotch viroid* (ASBVd) ribozyme (RZ) sequence was fused to the 3′ end to generate an authentic 3′ viral end in the transcribed mRNAs. The icDNA generated was mobilized into the *Agrobacterium tumefaciens* EHA 105, and the agrobacterial cultures were infiltrated into the natural host *C. papaya* and a non-host *Nicotiana benthamiana* plants; both did not show any symptoms. In RT-PCR analysis of RNAs isolated from *N. benthamiana*, we could detect viral genes as early as 3 days and continued up to 28 days post infiltration. Alternatively, virion particles were purified from agroinfiltrated *N. benthamiana* plants and introduced into *C. papaya* by mechanical inoculation as well as by pinprick method. In both cases, we could see visible systemic symptoms similar to that of wild type by 40 days. Additionally, we studied the expression patterns of the genes related to plant defense, transcription factors (TFs), and developmental aspects from both *C. papaya* and *N. benthamiana*.

## 1 Introduction

*Carica papaya* is an extensively cultivated, highly valued fruit crop due to its manifold nutritional and medicinal advantages (de Oliveira and Vitória, 2011). According to the FAO statistical database, 2020, India is the highest papaya-producing country in the world, with an annual yield of 5.7 million metric tonnes. However, like most crops, *C. papaya* farming is vulnerable to several pathogens, including bacteria, fungi, and viruses. Various viral pathogens from diverse families, such as *Potyviridae*, *Alphaflexiviridae*, *Geminiviridae*, *Tospoviridae*, and *Solemoviridae*, are commonly known to infect *C. papaya* plants. The *Potyviridae* family alone has 244 plant viruses classified into 12 genera, with most species belonging to the *Potyvirus* genus (Inoue-Nagata et al., 2022) infecting several economically significant crops, such as banana, beans, peanuts, chili, maize, watermelon, papaya, potato, and tobacco.

Papaya ringspot disease (PRSD) is the most destructive viral disease affecting *C. papaya* plants worldwide, as it poses a significant threat to the economies of the countries that produce papaya (Tripathi et al., 2008). The causative agent, *Papaya ringspot virus* (PRSV), is a *Potyvirus*, that can infect its host at any stage of growth in a systematic manner, resulting in a severe chlorotic and mosaic pattern on leaves, water-soaked streaks on leaf petioles and trunks, deformed fruits with ring-like spots, distorted and shoestring-like appearance of leaves along with reduced photosynthetic efficiency (Gonsalves et al., 2010). PRSV can infect *Caricaceae*, *Chenopodiaceae*, and *Cucurbitaceae* family members experimentally. Based on the host range, it has two subtypes: PRSV-P and PRSV-W (Yeh, 1984). More than 24 species of aphids are involved in the transmission of PRSV in a non-persistent manner, with *Myzus persicae*, *Aphis gossypii*, and *A. craccivora* being the most efficient ones (Kalleshwaraswamy and Kumar, 2008). The PRSV genome is comprised of a 10.3 kb positive-sense single-stranded RNA containing a single open reading frame (ORF), which encodes a single large polyprotein and a ribosomal frameshifting product PIPO (Chung et al., 2008; Gonsalves et al., 2010). The polyprotein gets cleaved into 10 different mature proteins by three of its own proteases: P1 pro, HC-Pro, and NIa-Pro (Yeh et al., 1992; Tripathi et al., 2008).

Infectious cDNA clones of plant viruses have been proven to be highly effective tools for the confirmation of Koch’s postulates, reverse genetic approaches, selecting plant in-breeding programs, and investigating the intricate interaction between viruses and their hosts (Navas-Hermosilla et al., 2021). This approach offers invaluable insights into the viral life cycle and pathogenesis, ultimately facilitating a better understanding of such processes (Boyer and Haenni, 1994). Since the inception of the first infectious clone of the *Brome mosaic virus* (BMV) (Ahlquist et al., 1984), agrobacterium-mediated icDNAs have been engineered for several plant viruses, paved the way for molecular manipulations and functional characterization studies *in planta* and greatly expanded the scope of research in this field (Díaz-Cruz et al., 2018).

The study of plant viruses has been primarily directed toward their detrimental impact on crop plants (Anderson et al., 2004; Nelson and Citovsky, 2005). To safeguard themselves from these threats, plants employ pre-existing defense mechanisms to recognize elicitor(s) linked to the attacker and initiate the appropriate defense responses (Zhou and Zhang, 2020). However, many viruses encode RNA-silencing suppressor proteins to prevent the recognition of small interfering RNA (siRNA), inhibiting the RNA-silencing pathway (Zhao et al., 2016). Plant hypersensitive response against abiotic stresses includes the formation of reactive oxygen species (ROS), induction of salicylic acid (SA) and jasmonic acid (JA) signaling, and *PR* gene response (Akbudak et al., 2020). Transcription factors (TFs) are highly sought after for genetic engineering because they regulate stress-related genes (Baillo et al., 2019). Manipulating TFs has become a popular research topic, as many respond to stress and control several downstream genes, making them promising candidates for improving plant stress tolerance (Hoang et al., 2017). Many TF families, such as MYB, bZIP, NAC, ERF, and many more, have been identified to be involved in biotic stress response in plants (Ng et al., 2018).

Viral infections significantly impact plant growth, regenerative ability, and physiological metabolism (Jiang and Zhou, 2023). A recent study highlighted the crucial role of cytokinin(s) in regulating the transcriptional expression of downstream genes during various stages of leaf development (Wu et al., 2021). Jasmonates (JA) is another type of phytohormone that regulates defenses against herbivores and pathogens and plays a crucial role in plant development (Goossens et al., 2016). When viruses invade plants, they undergo various physiological and molecular changes, including stunted growth, poorly developed leaves, chlorophyll degradation and consequently leaf senescence, programmed cell death (PCD), and autophagy (Espinoza et al., 2007; Park et al., 2007; Tang and Bassham, 2018; Wu et al., 2021).

The unavailability of a significant number of complete PRSV genome sequences from different geographical regions of India has been a substantial limitation in understanding the molecular basis of papaya ringspot disease severity at the national and global levels. It is essential to surveil the circulating PRSV strains to keep track of the genotypic and phenotypic traits of the host plant. A comparative study regarding the agroinfectivity of the infectious cDNA in the natural host and non-host plants was conducted to monitor plant response(s) based on the expression change of essential plant genes and TFs.

## 2 Materials and methods

### 2.1 Field survey and virus isolate

During field surveys, a papaya orchard was identified at the Shankarpalli mandal (17° 28′ 7.04″ N, 78° 7′ 54.16″ E) of Rangareddy district, Telangana, India, where the leaves of several plants appeared yellowish, distorted and shoestring-like. The leaf samples from different plants of that orchard were collected and brought to the laboratory for further analysis. The presence of PRSV was confirmed by performing DAC-ELISA and RT-PCR analysis (Supplementary Figure S1A). Based on these analyses, one sample (Shankarpalli 4) was chosen for further analysis.

### 2.2 Total RNA extraction, cDNA synthesis, and cloning of cDNA fragments

**Figure 1:**
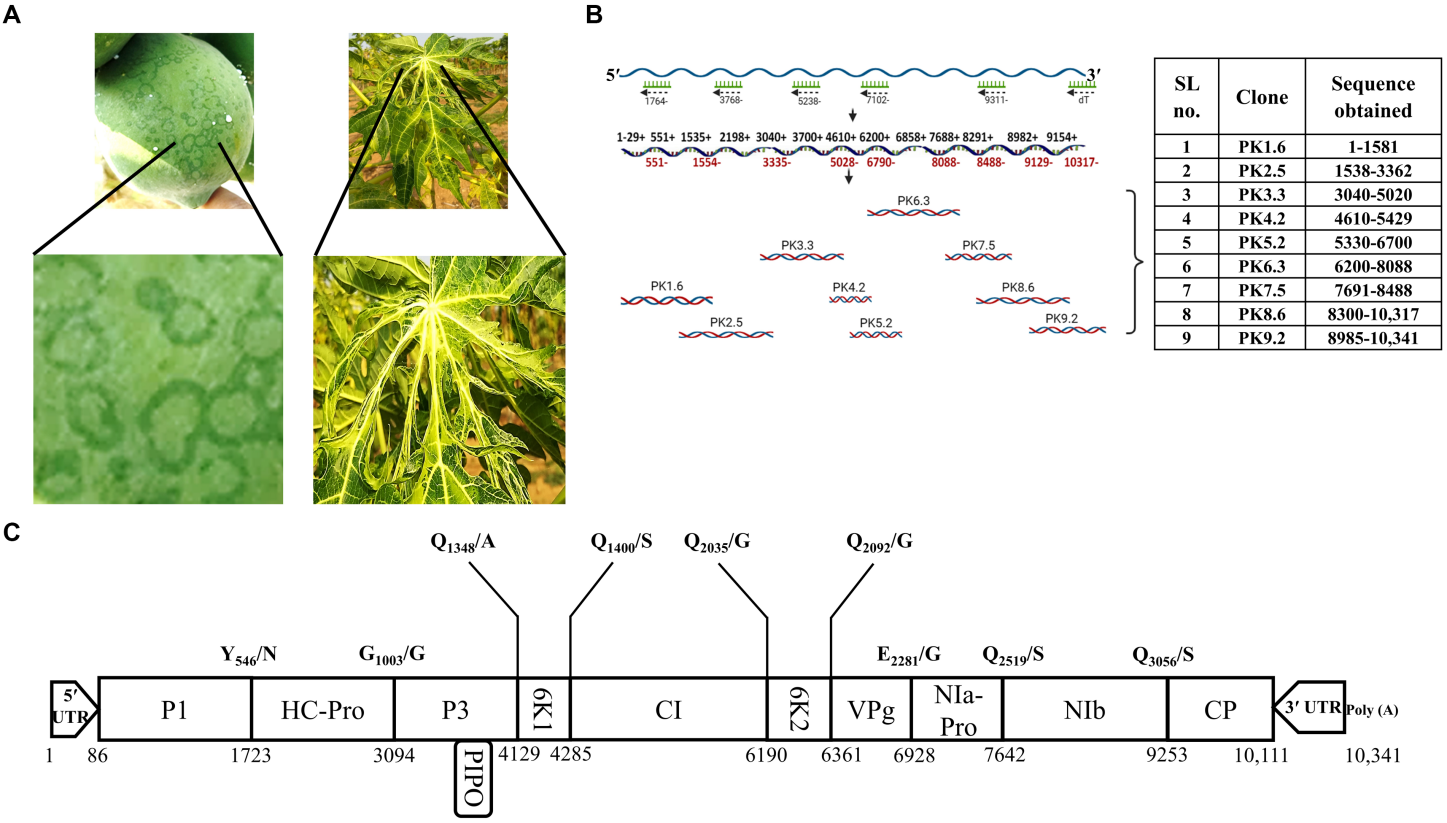
Symptomatology, complete genome sequencing, and the putative genome organization of PRSV-HYD isolate. **(A)** The infected leaf and fruit samples were collected based on the visible symptoms- ringspots on fruits and distorted leaves. **(B)** The full-length PRSV-HYD genome was converted into nine overlapping cDNA fragments, amplified, and cloned into the pGEM-T Easy vector (Promega). These nine TA clones were sequenced on both strands, with three biological replicates of each. Sequences obtained from each clone are as follows: PK1.6- nucleotide 1–1,581, PK2.5–1,538-3362, PK3.3–3,040-5020, PK4.2–4,610-5429, PK5.2–5,330-6700, PK6.3–6,200-8088, PK7.5–7,691-8488, PK8.6–8,300-10,317, and PK9.2–8,985-10,341. **(C)** The deduced genomic map of PRSV-HYD with the exact position of individual genes and putative cleavage sites of the proteins.

Total RNA was isolated from the infected leaf samples using TRIzol® reagent (Invitrogen) following the manufacturer’s instructions. First-strand cDNA synthesis was carried out using primers designed (Supplementary Table S1) based on the complete genome sequence of the PRSV-Del isolate (Accession no. EF017707.1) with the help of SuperScript™ III Reverse Transcriptase (Invitrogen). This cDNA mixture was diluted up to 50 μL to amplify the full-length viral genome in overlapping fragments using several combinations of sense and antisense primers and *Taq* DNA polymerase (NEB). The PCR products were purified using a QIAquick gel extraction kit (QIAGEN) and ligated in pGEM-T Easy (Promega) TA cloning vector following the manufacturer’s instructions. Nine cDNA clones spanning the entire genome length were designated PK1.6- PK9.2 and selected for sequencing analysis (Figure 1B).

### 2.3 Complete genome sequencing and *in silico* analysis of PRSV-HYD

To obtain the complete genome sequence of PRSV-HYD, we sequenced a minimum of 3 positive clones for each of the nine cDNA clones on both strands with the help of sequencing primers (Supplementary Table S1) using the Sanger’s dideoxy method by 3,500 XL Genetic analyzers (ThermoFisher Scientific). Authentic 5′- and 3′- terminal sequences of PRSV cDNA were confirmed by using SMARTer® 5′ RACE and 3′ RACE kits (TaKaRa Bio) by following the manufacturer’s instructions. A minimum of 5 independent clones were sequenced to confirm the termini. The obtained raw sequences from 9 different pGEM-T easy clones were trimmed and assembled using PRSV-Del (Accession no. EF017707.1) genome sequence as a template. To identify ORFs, the ORF finder tool^1^ was used. *In silico* translation was performed using the translate tool by ExPASy (Gasteiger et al., 2003). The full-length nucleotide sequence and the *in silico* translated polyprotein sequences were used for blastn and blastp analysis, respectively. This helped us obtain closely related PRSV sequences from the NCBI database based on sequence homology. Phylogenetic analysis was carried out using MEGA-X version 10.1.8 with the Maximum-likelihood method with the Jones-Taylor-Thornton (JTT) model (for polyprotein sequences) with 1,000 bootstrap replicates (Kumar et al., 2018).

### 2.4 Infectious cDNA (icDNA) construction for PRSV-HYD

**Figure 2:**
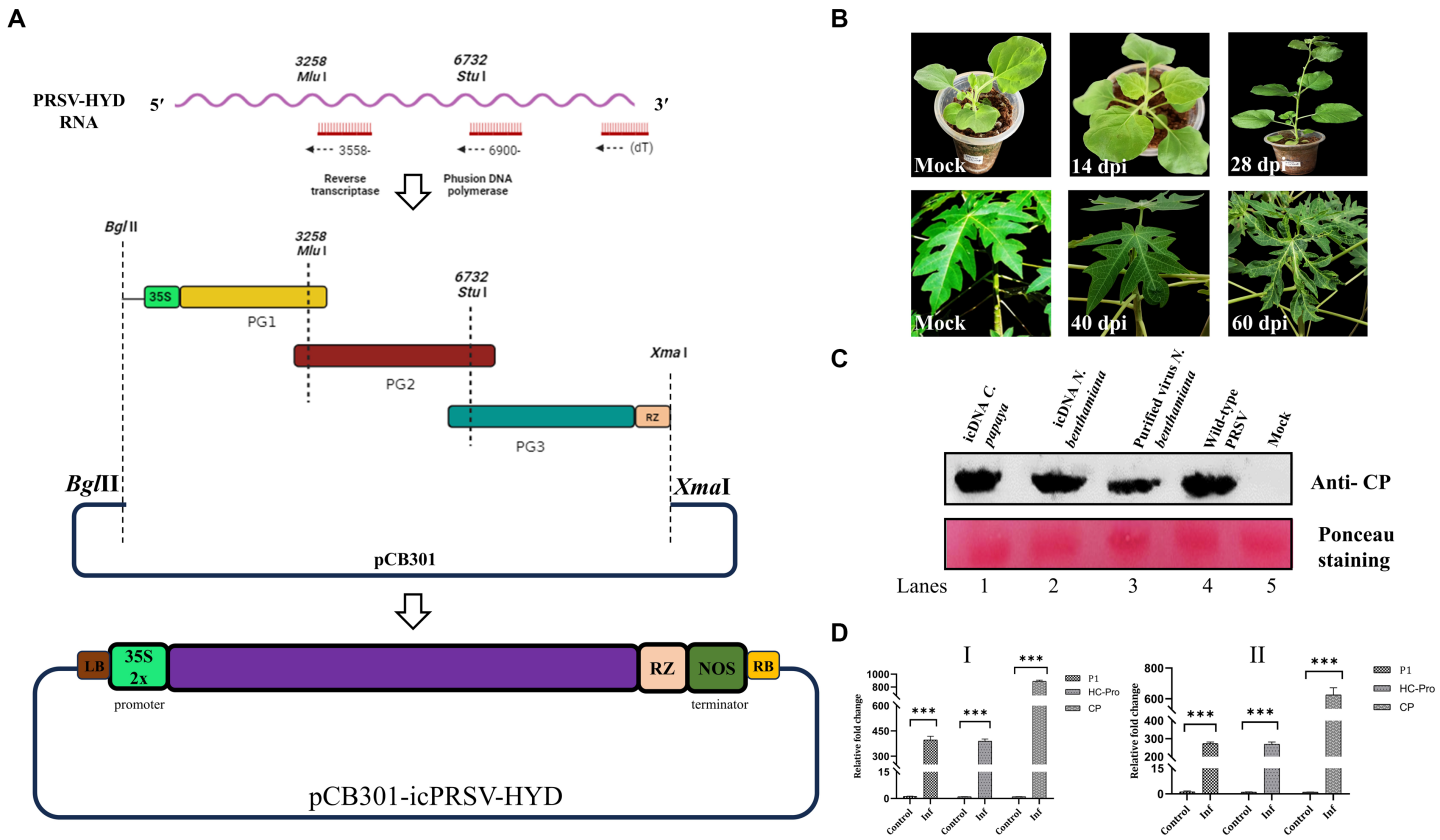
PRSV-HYD infectious clone preparation and its infectivity in different hosts. **(A)** The schematic representation of the PRSV-HYD infectious clone preparation; three sequence-specific primers were used for reverse transcription, followed by amplification using Phusion DNA polymerase (NEB). *Bgl*II restriction site followed by CaMV 35S double promoter was fused to the 5′ terminus by primer overlap extension PCR. Similarly, the ASBVd RZ sequence followed by the *Xma*I restriction site was added to the 3′ terminus. Then, restriction digestion was performed using *Bgl*II-*Mlu*I (PG1), *Mlu*I*-Stu*I (PG2), and *Stu*I-*Xma*I (PG3). These three double-digested fragments were ligated into the pCB301 vector backbone. **(B)** *Carica papaya* leaves showed mild leaf distortion symptoms at 40 dpi, and the symptom severity was observed at 60 dpi. In the case of *N. benthamiana*, no symptoms were observed on the inoculated leaves (14 dpi) or systemic leaves (28 dpi). Empty vector pCB301 in *A. tumefaciens* served as control. **(C)** Immunoblot analysis using PRSV coat protein (CP) specific antibody showed expression of CP in *C. papaya* leaves (60 dpi) inoculated with the purified virus particles from agroinfiltrated *N. benthamiana* plants (Lane-1), agroinfiltrated *N. benthamiana* leaves (28 dpi) (Lane-2), and purified virus particles from the agroinfiltrated *N. benthamiana* plants (28 dpi) (Lane-3). Wild-type PRSV-infected *C. papaya* leaves served as a positive control (Lane-4), and empty vector pCB301 in *A. tumefaciens* served as mock (Lane-5). **(D)** Real-time PCR analysis showed significant upregulation of *P1*, *HC-Pro*, and *CP* genes in both *C. papaya* (I) and *N. benthamiana* (II) plants.

To construct PRSV-HYD icDNA, TRIzol (Invitrogen)-extracted total RNA was sequentially reverse transcribed using SuperScript™ III Reverse Transcriptase (Invitrogen) and three sequence-specific primers (Supplementary Table S1) as shown in Figure 2A. Further, using Phusion™ High-Fidelity DNA Polymerase (NEB), the cDNAs were converted into three overlapping PCR amplified fragments designated as PG1, PG2, and PG3 (Figure 2A), encompassing the full-length PRSV-HYD genome with a minimum of 50 nucleotide overlap. By performing primer overlap extension PCR, *Cauliflower mosaic virus* (CaMV) 35S double promoter sequence containing *Bgl*II restriction site was fused to the 5′ end of PG1 fragment. Similarly, a ribozyme (RZ) sequence from *Avocado sunblotch viroid* (ASBVd) followed by *Xma*I restriction site was added at the 3′ end of PG3 fragment (Gopinath et al., 2005). PG1, PG2, and PG3 fragments were double digested with *Bgl*II-*Mlu*I, *Mlu*I-*Stu*I, and *Stu*I-*Xma*I, respectively. The fragments were gel-purified using a QIAquick gel extraction kit (QIAGEN) and ligated into the vector backbone (binary vector pCB301) digested with *Bgl*II/*Xma*I (Figure 2A). The ligation mix was transformed into competent ElectroMAX™ Stbl4™ cells (Invitrogen) by electroporation (25 μF, 200 Ω, 1200 V) in a Gene pulser XCell (Bio-Rad). A total of six clones were confirmed by colony PCR and restriction digestion. All six positive clones were sequenced on both strands using sequence-specific primers and matched entirely with the full-length PRSV-HYD sequence. A minimum of three positive clones (pCB301-icPRSV-HYD) were mobilized into the *Agrobacterium tumefaciens* EHA105 strain by electroporation using the parameters mentioned above.

### 2.5 Agroinoculation of plants

Agroinoculation of plants with pCB301-icPRSV-HYD in *A. tumefaciens* EHA105 cells was carried out following the method described by Gopinath et al. (2005). Briefly, *A. tumefaciens* EHA105 cells containing pCB301-icPRSV-HYD were allowed to grow in Luria broth (LB) supplemented with Rifampicin (50 μg/mL) and Kanamycin (50 μg/mL) at 28°C with 180 rpm of continuous shaking. 1 mL of the pre-culture was used to inoculate 100 mL of LB-Rifampicin-Kanamycin supplemented freshly with 10 mM sterile 2-(N-Morpholino) ethanesulphonic acid (MES) pH 5.85 and 20 mM of acetosyringone, and the culture was grown for 36 h at 28°C incubator with 180 rpm shaking. Cells were harvested by centrifugation at 6000 rpm for 10 min and then resuspended in 10 mL of infiltration buffer (10 mM MgCl_2_ and 10 mM MES pH 5.85) with 100 μM acetosyringone. This suspension was incubated at room temperature for 3 h. *A. tumefaciens* carrying empty vector pCB301 served as a negative control. The suspension was diluted to OD_595_ 0.1 with infiltration buffer and infiltrated at the abaxial surface of 25 *Nicotiana benthamiana* plants using a 2 mL syringe without a needle. Infiltrated leaves were harvested from all these plants at a regular interval of 3 days. Then, virion particles were purified from this batch of *N. benthamiana* plants at 28 dpi according to the method explained by Moghal and Francki (1976). The purified virus particles were mechanically inoculated onto the *C. papaya* leaves (3-leaf stage) and pinpricked the young stems by sterile needles (Zhang et al., 2021). All the agro-inoculated plants were maintained inside an insect-free climate chamber (25°C/21°C Day/night temperature, 70% relative humidity, 16 h photoperiod at 150 μmol s^−1^ m^−2^ light intensity).

### 2.6 Analysis of agro-inoculated icDNAs in *Carica papaya* and *Nicotiana benthamiana*

To check the functionality of the icDNA, two approaches were used. Initially, total RNAs were isolated from *N. benthamiana* leaves infiltrated with PRSV icDNA from 3 to 28 days at regular intervals. RT-PCRs were performed for P1, HC-Pro, and CP genes individually. However, we have relied on the development of symptoms in the case of *C. papaya*. Secondly, total soluble proteins were isolated from the *N. benthamiana* and *C. papaya* following the protocol described by Gopinath et al. (2000). Immunoblot analysis was performed using PRSV CP-specific antiserum using chemiluminescent detection system (Thermo Fisher Scientific) according to the manufacturer’s instructions.

### 2.7 Quantitative analysis of virus and host genes from *Nicotiana benthamiana* and *Carica papaya*

Total RNA isolated from the agroinfiltrated plants were quantified using NanoDrop 2000 UV–Vis Spectrophotometer (ThermoFisher Scientific). Primers targeted for the genes encoding three viral proteins (P1, HC-Pro, and CP) and host genes encoding pathogenesis-related gene-1 and 10 (*PR1a* and *PR10*), defensin-like protein 1.2 (*PDF1.2*), Ran-binding protein 1a (*RanBP1*); host transcription factors basic leucine zipper 60 (*bZIP60*), *NbNAC042*, Ethylene responsive transcription factor-5 (*ERF5*), *MYB44*, Isopentenyl transferase-1 (*IPT1*), Lonely Guy-1 (*LOG1*), Allene oxide cyclase-1 (*AOC1*), 12- oxo-phytodienoic acid reductase-2 (*OPR2*), Bax inhibitor-1 (*BI-1*) Staygreen1 (*SGR1*), senescence-associated gene-12 (*SAG12*), autophagy 8f (*ATG8f*) were synthesized using GenScript (Supplementary Table S2). cDNA was synthesized from the total RNA, as described earlier. We performed Real-time PCR on Mastercycler Realplex (Eppendorf, Germany), as reported previously (Supriya et al., 2022). The actin genes of *C. papaya* and *N. benthamiana* (AY179605.1) were used as an internal controls for respective plants. The relative expression levels of the undertaken genes were estimated using the ΔΔC_T_ method (Livak and Schmittgen, 2001). Three biological replicates were used for every individual gene.

### 2.8 Statistical analysis

The data presented represents the mean value of three treatments, each containing three replicates. The data was analyzed using a two-way ANOVA. The error bars on the graph represent the standard deviation (± SD) of the mean values. The Duncan multiple range test (*p* ≤ 0.05) was used to determine significant treatment differences.

## 3 Results

### 3.1 Complete genome sequencing of PRSV-HYD

In this study, we collected *C. papaya* leaves and fruits suspected to be infected with PRSV (Figure 1A). The presence of PRSV was confirmed by DAC-ELISA and RT-PCR analysis (Supplementary Figure S1). We generated nine overlapping PCR amplified fragments by performing RT-PCR using different combinations of primers, followed by successful TA cloning of these fragments. Positive clones were confirmed through colony PCR (data not shown). The positive clones were designated as PK1.6, PK2.5, PK3.3, PK4.2, PK5.2, PK6.3, PK7.5, PK8.6, and PK9.2 (Figure 1B). From the sequencing data of these nine overlapping TA clones (Figure 1B), complete genome sequence was deduced (Figure 1C). We determined the terminal nucleotide sequences using 5′- and 3′- RACE techniques (Supplementary Figure S2). The complete genome is 10,341 nucleotides in length. A single large putative ORF was identified, starting from the 86^th^ nucleotide and ending at the 10,111^th^ nucleotide of the genome. Putative sizes of the coding and non-coding regions were identified (Table 1). 3,342 amino acids long polyprotein sequence was identified by *in silico* analysis. We identified the putative cleavage sites of the functional proteins (Figure 1C) by aligning them with other PRSV sequences from the NCBI GenBank database. We deposited the deduced full-length genome sequence in the NCBI GenBank database with accession number KP743981.1. We have also identified conserved regions based on previous reports of the known potyviruses available in the NCBI databases (Supplementary Figure S3). Some of those notable conserved motifs are G_496_SSG in P1, F726RNK in HC-Pro, N_2868_GDDL in NIb, D_3063_AG in CP, and a stretch of KE amino acids at the N-terminus of the CP.

**Table 1:** Putative sizes of individual genes and mature proteins of PRSV-HYD were identified and documented.

Genomic region	Size in bp (amino acids)	% Sequence identities with different PRSV strains Asian	American	European
Whole genome	10,341/ (−)	81–92	86–88	87–88
Polyprotein	−/ (3342)	(87–95)	(91–92)	(92)
5′ UTR	85/ (−)	79–100 (−)	79–100 (−)	79 (−)
P1	1,638/ (546)	67–89 (59–87)	75–78 (71–75)	77 (74–75)
HC-Pro	1,371/ (457)	84–94 (92–98)	89–91 (96–97)	91 (96–97)
P3	1,035/ (345)	81–92 (88–95)	88–90 (92–94)	89 (93)
6 K1	156/ (52)	81–92 (77–96)	85–92 (90–100)	88 (90)
CI	1905/ (635)	80–94 (93–98)	90–92 (97–98)	91–92 (98–99)
6 k2	171/ (57)	80–92 (87–95)	86–92 (89–96)	90–91 (91)
VPg	567/ (189)	80–94 (92–97)	87–91 (93–97)	91 (96)
NIa-Pro	714/ (238)	80–93 (91–97)	88–91 (94–96)	89 (95)
NIb	1,611/ (537)	81–93 (89–95)	88–90 (92–95)	89 (96)
CP	858/ (286)	85–95 (85–97)	86–88 (87–92)	87–88 (94)
3′ UTR	230/ (−)	88–94 (−)	90–94 (−)	91–92 (−)

Pairwise percent nucleotide and amino acid sequence identities of different genomic regions of PRSV-HYD compared with other PRSV isolates from Asian, American, and European countries are shown. Protein size and amino acid sequence identities are shown in parentheses. PRSV-HYD P1 has been identified as the most diverse genomic region.

### 3.2 Phylogenetic analysis of PRSV-HYD

**Figure 3:**
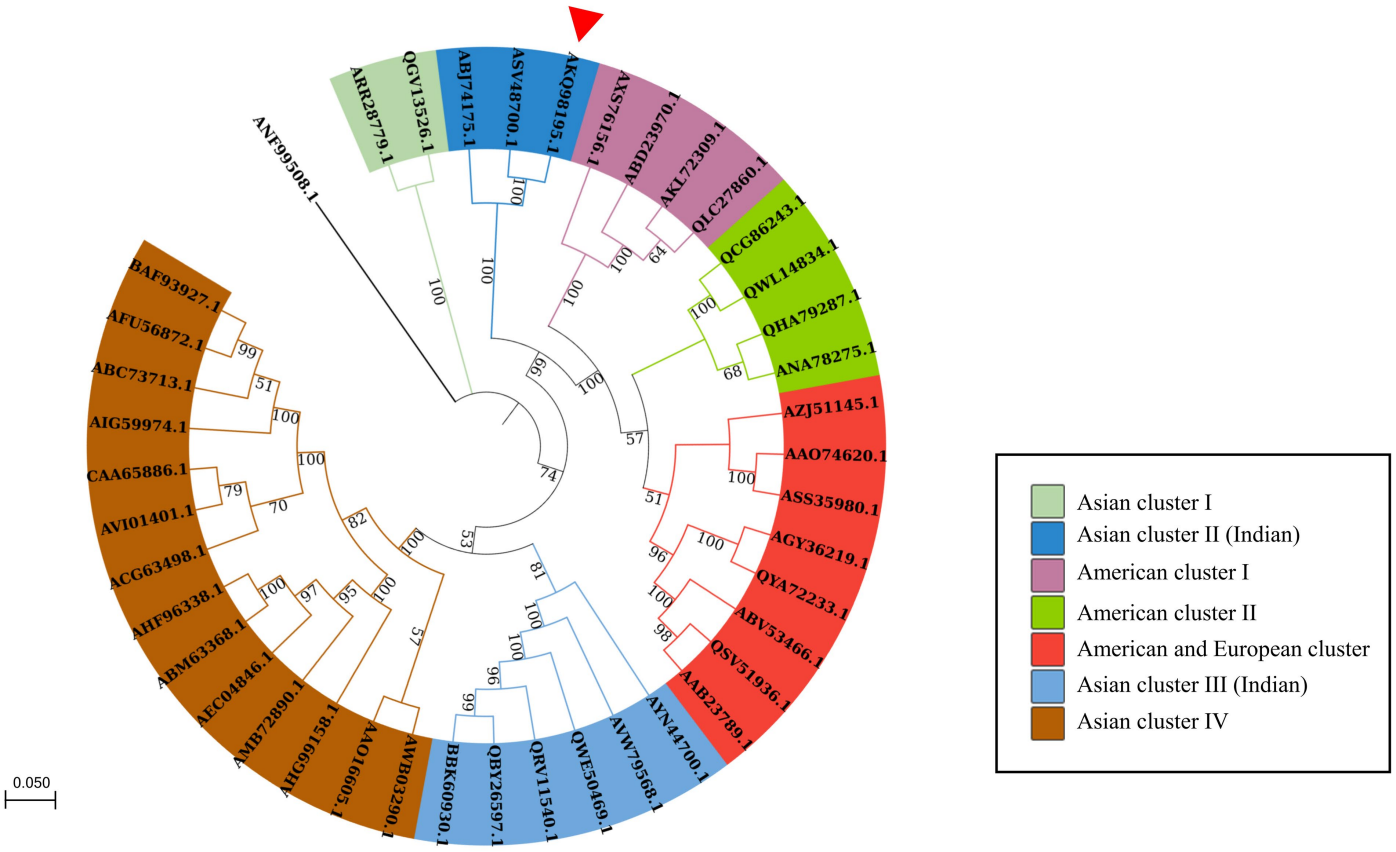
Phylogenetic analysis of the polyprotein sequence of PRSV-HYD isolate (accession no. AKQ98195.1) along with 40 closely related PRSV polyprotein sequences retrieved from the NCBI database and a *Moroccan watermelon mosaic virus* (accession no. ANF99508.1) isolate as an outgroup. The sequences were aligned using ClustalW, and a maximum-likelihood phylogenetic tree was constructed with MEGA-X [14]. The numbers at the nodes represent the percentage of 1,000 bootstraps. Bootstrap values less than 50% are not shown. PRSV-Pune (Aundh) and PRSV-Del isolate (accession no. ASV48700.1 and ABJ74175.1) are present within the same clade as PRSV-HYD, with PRSV Pune isolate being the closest neighbor to PRSV-HYD, has been shown with a red triangle.

A maximum likelihood tree was created with 1,000 bootstrap replicates using the *in silico*-translated PRSV-HYD polyprotein sequence (Accession no. AKQ98195.1) and other available PRSV polyprotein sequences from different regions worldwide. PRSV Pune (Aundh) and PRSV-Del isolates (Accession no. ASV48700.1 and ABJ74175.1, respectively) were present within the same clade as PRSV-HYD, with PRSV Pune (Aundh) being the closest neighbor. The isolates have been grouped into distinct clusters based on their geographical locations, and individual clusters have been denoted with distinct color codes (Figure 3).

Another maximum likelihood tree was constructed with 1,000 bootstrap replicates involving PRSV-HYD and 25 other Potyviral polyprotein sequences. *Zucchini tigre mosaic virus* (ZTMV) USA isolate (ATY37425.1), *Papaya leaf distortion mosaic virus* (PLDMV) Hainan isolate (AGC54443.1), and *Cucurbit vein banding virus* (CVBV) Argentina isolate (ASB15795.1) and PRSV-HYD (AKQ98195.1) are present within the same clade. ZTMV is the closest neighbor to PRSV-HYD (Supplementary Figure S4).

Furthermore, PRSV-HYD complete nucleotide and amino sequence shows up to 92 and 95% of sequence homology, respectively, with other Asian isolates. The 5′- non-coding region and P1 coding region showed the highest sequence diversity of up to 21 and 33%, respectively, which is the maximum compared to other genomic regions. P1 amino acid sequence showed up to 41% of sequence diversity compared to the Asian isolates (Table 1).

### 3.3 Construction of PRSV-HYD icDNA and its expression patterns *in planta*

To Construct PRSV-HYD icDNA, the three amplified cDNA fragments PG1, PG2, and PG3 were (3.7 kb, 3.2 kb, and 3.4 kb in size, respectively) used. By employing diverse molecular manipulations, PRSV-HYD infectious cDNA was constructed in the binary vector pCB301 under the right and left borders of the T-DNA region. *Cauliflower mosaic virus* 35S double promoter was fused at the 5′ end of the genome for efficient transcription. Care was taken that no additional sequences are introduced in the transcribed RNAs. Similarly, at the 3′ end, *Avocado sunblotch viroid* RZ sequence was fused for *cis* preferential cleavage and generation of authentic viral ends. The resultant clones were sequence-confirmed and designated as pCB301-icPRSV-HYD (Figure 2A). Upon agroinfiltration, no visible symptoms were observed up to 28 dpi in *N. benthamiana* plants, indicating this could be an asymptomatic host. However, RT-PCR analysis demonstrated the presence of *P1*, *HC-Pro*, and *CP* gene products in all the *N. benthamiana* plants infiltrated from 3 dpi up to 28 dpi (Supplementary Figure S5). This is clear evidence of the efficient transcription of the icDNAs introduced under the CaMV 35S double promoter.

We have not observed any visible symptoms up to 120 dpi in *C. papaya* plants infiltrated with icDNAs. The plants are not as conducive to infiltrations as *N. benthamiana*. Alternatively, we have purified virus particles from *N. benthamiana* plants (28 dpi) infiltrated with the icDNAs using the protocol of Moghal and Francki (1976). This purified virus was introduced into the *C. papaya* plants by mechanical inoculation, and the virus particles were introduced into the young stems by the pinprick method.14 out of 25 mechanically inoculated plants and 20 out of 25 plants inoculated by the pinprick method, started showing symptoms 40 days post-inoculation, indicating the infectivity of infiltration-generated virus particles and the symptom severity increased by 60 dpi (Figure 2B). These results were confirmed in immunoblot analysis using PRSV-Del CP polyclonal antibody (Figure 2C). The results were further substantiated in Real-time PCR, where we observed the upregulation of the three viral gene products *CP*, *P1*, and *HC-Pro* in both *N. benthamiana* and *C. papaya* (Figure 2D). The newly emerging leaves of both *C. papaya* and *N. benthamiana* were analyzed for the presence of viral CP gene by RT-PCR analysis to confirm the systemic movement of the virus particles. We could detect the PRSV CP gene in the systemic leaves of *C. papaya* indicating its systemic spread beyond the infiltrated leaves (Supplementary Figure S6A). However, we failed to detect PRSV CP gene from the systemic leaves of *N. benthamiana* indicating its inability to go systemic (Supplementary Figure S6B).

### 3.4 Comparison of host-specific gene expression patterns between natural host and non-host of PRSV-HYD

**Figure 4:**
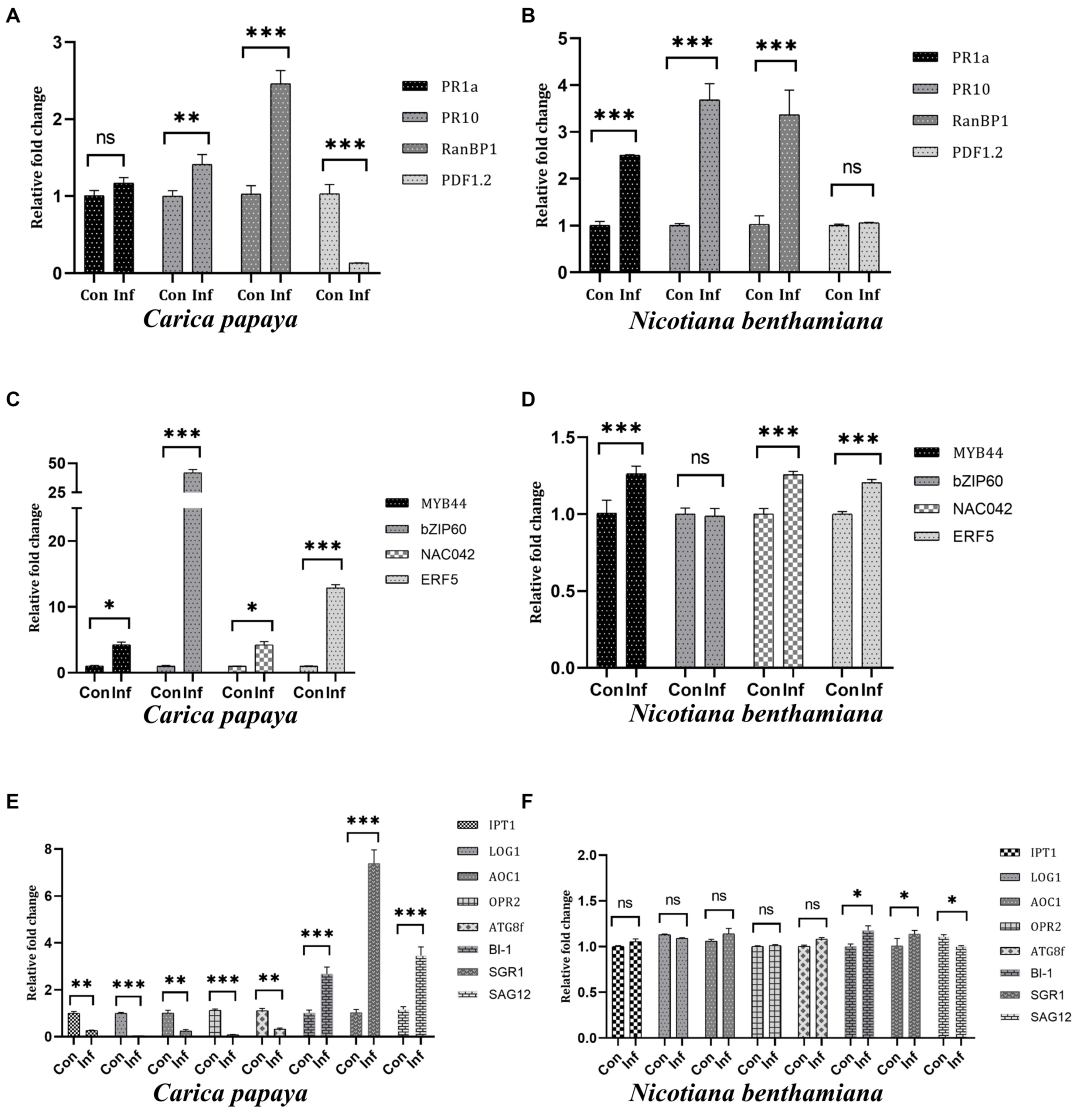
Relative expression levels of different plant-specific genes from host (*C. papaya*) **(A,C,E)** and non-host (*N. benthamiana*) **(B,D,F)** plants. **(A)** It was observed that the genes *PR1a* showed no change, whereas *PR10* and *RanBP1* showed 1.4-fold and 2.4-fold upregulation. Meanwhile, *PDF1.2* was significantly downregulated. **(B)** Significant upregulation of *PR1a*, *PR10*, and *RanBP1* was observed in non-host plants, while *PDF1.2* expression remained at a regular level. **(C)** All four TFs were found to be upregulated in host plants, with *bZIP60* showing the highest upregulation of 42-fold. **(D)** *MYB44*, *NAC042*, and *ERF5* showed 1.26-, 1.25- and 1.20-fold upregulation in expression levels in non-host plants, while *bZIP60* was expressed at a regular level. **(E)** Cytokinin biosynthesis-related genes *IPT1* and *LOG1*, Jasmonate biosynthesis genes *AOC1* and *OPR2*, and autophagy-related gene *ATG8f* were significantly downregulated in host plants, while *BI-1*, *SGR1*, and *SAG12* genes were upregulated significantly. **(F)** There were no significant changes in the expression levels of *IPT1*, *LOG1*, *AOC1*, *OPR2*, and *ATG8f* genes in non-host plants. *BI-1* and *SGR1* showed a 1.17- and 1.13-fold change, while *SAG12* showed no change in expression.

All the real-time PCR analyses were performed using total RNA extracted from 28 dpi *N. benthamiana* and 60 dpi *C. papaya* plants. In *C. papaya*, *PR1a* showed no change, *PR10* and *RanBP1* were upregulated by 1.4-fold and 2.4-fold, respectively, whereas *PDF1.2* was significantly downregulated (Figure 4A). *PR1a*, *PR10*, and *RanBP1* genes were significantly upregulated in *N. benthamiana*, but *PDF1.2* showcased unchanged expression compared to control plants (Figure 4B). The expression levels of some transcription factors, such as MYB44, basic Leucine zipper-60 (bZIP60), NAC042, and Ethylene response factor-5 (ERF5), were checked. In the case of *C. papaya*, all four transcription factors showed significant upregulation, with *bZIP60* and *EFR5* showing the highest level of upregulation by 42- and 13-fold, respectively (Figure 4C). *N. benthamiana* showed 1.26-, 1.25- and 1.20-fold upregulation of *MYB44*, *NAC042*, and *ERF5*, respectively, but there was no change in *bZIP60* expression (Figure 4D). Some genes that are directly related to the physiology of plants, such as Isopentenyl transferase-1 (*IPT1*), Lonely Guy-1 (*LOG1*), Allene oxide cyclase-1 (*AOC1*), 12- oxo-phytodienoic acid reductase (*OPR2*), and Autophagy-related protein 8f (*ATG8f*) were also checked. The host plants showed significant downregulation of all these five genes, whereas the non-host *N. benthamiana* exhibited no change in expression levels. When we checked the expression levels of Bax inhibitor-1 (*BI-1*), Staygreen-1 (*SGR1*), and senescence-associated gene-12 (*SAG12*), these genes were found to be upregulated by 2.6, 7.3, and 3.4-fold, respectively in *C. papaya* (Figure 4E). On the other hand, *BI-1* and *SGR1* showed a 1.17- and 1.13-fold change in *N. benthamiana*, whereas *SAG12* showed 1.1-fold downregulation (Figure 4F).

## 4 Discussion

The severity of PRSV infection in papaya orchards is rising alarmingly with time (Premchand et al., 2023). The lack of availability of a significant number of full-length genomic sequences is one of the major drawbacks in viral disease management. We sequenced and confirmed PRSV infection in the samples from an orchard mentioned earlier (Figure 1). This full-length genome sequence provides us with information about the diverse nature of this virus, conserved regions or motifs, mutation-prone genomic regions, and many more. The presence of some of the conserved motifs is essential for successful PRSV infection, as different motifs have different functions such as GDSG motif is required for the protease activity of P1 (Adams et al., 2005), FRNK within HC-Pro is involved in symptom development (Gal-On, 2000), GDD motif of NIb is important for RNA-dependent RNA polymerase activity (Liu et al., 2021), coat protein DAG motif is required for aphid transmission (Bravo et al., 2008), a region of KE repeats at the N-terminal region of coat protein with unknown function (Shukla et al., 1994), and many others. All these conserved motifs with minor modifications have been identified in PRSV-HYD, and the modifications could be due to the natural diversities that did not hinder the functionality. P1 is the most diverse functional protein among potyviruses (Tordo et al., 1995; Mishra et al., 2019). Our study showed the same outcome, with P1 showing maximum nucleotide and amino acid sequence diversities compared to other PRSV isolates. The 5′ UTR was also more diverse than the 3′ UTR (Table 1). We can presume that the 5′ terminal of the PRSV genome might be more prone to random mutations than the 3′ terminal. This complete genome sequence data will contribute to the further understanding of PRSV epidemiology and sequence diversity studies.

ZTMV, PLDMV, CVBV, and PRSV-HYD sharing the same clade signifies their similar evolutionary origin. ZTMV-USA isolate (Accession no. ATY37425.1) is the closest to PRSV-HYD, and these two isolates share 67.2% of amino acid sequence homology, suggesting they might have a common or closely related ancestor. PRSV Pune (Aundh) (Accession no. ASV48700.1) is the closest relative to PRSV-HYD, with a 95% amino acid sequence similarity, suggesting they might have a similar evolutionary origin (Figure 3).

The importance of having stable infectious cDNA clones for any RNA virus is well-known in the field of plant virology. Several infectious clones available for many potyviruses, such as *Watermelon mosaic virus* (WMV) (Desbiez et al., 2012), *Potato virus* Y (PVY) (Chikh Ali et al., 2011), *Tobacco vein mottling virus* (TVMV) (Domier et al., 1989), *Zucchini yellow mosaic virus* (ZYMV) (Gal-On et al., 1991), *Soybean mosaic virus* (SMV) (Bao et al., 2020), *Plum pox virus* (PPV) (Riechmann et al., 1990), and many more. We have successfully developed icDNA for PRSV-HYD, which was agroinfectious in *N. benthamiana*; however, no visible symptoms appeared in the infiltrated plants, indicating this as an asymptomatic host (Figure 2). We have some limitations in the infiltration protocol of *C. papaya*. However, the infiltration-generated virus particles from *N. benthamiana* were infecting the *C. papaya* plants efficiently, with systemic movement of the virus particles. As *C. papaya* is the natural host of PRSV-P, there might be certain essential factors that are present only in *C. papaya* plants that aid in the establishment and multiplication of PRSV. These factors may play a crucial role in promoting PRSV systemic infection. So, the absence of such factors can be a reason for non-systematic infection in *N. benthamiana*.

The importance of PR genes is invaluable regarding the host response during any biotic or abiotic stress condition. *PR1a* gene is an activator of the Salicylate (SA) pathway and, thereby, plays an essential role in systemic acquired resistance (SAR) (Ali et al., 2018). *PR10* genes have been shown to possess anti-microbial activities during biotic stress (Chen et al., 2006). *RanBP1* is another crucial gene of plants as it accumulates terpenoids that show resistance against several pathogens (Mizuno et al., 2019). The expression levels of the *PR* genes and *RanBP1* were relatively lesser in the case of *C. papaya* plants compared to that in *N. benthamiana*, indicating that the natural host resistance is not strong enough against PRSV-HYD to overcome its symptoms. However, real-time PCR and immunoblot analysis proved that the virus replicates inside the non-host. However, the non-host plant has exerted resistance against the invasion of the virus, but the virus can still replicate without any symptoms. *PDF1.2* is a gene of the plant defensin family. It is known to be a part of the innate immune system and is involved in jasmonate (JA)- dependent defense response (Manners et al., 1998; Brown et al., 2003). Downregulation of *PDF1.2* in the host but unchanged in the non-host can be described as the ability of the virus to overcome the innate immune barrier of the natural host but not the non-host (Figures 4A,B).

Transcription factors (TFs) are essential as they regulate several genes involved in different plant growth and development stages, metabolic pathways, and many other molecular events. MYB44 TF gets activated by ethylene, which has multiple roles in growth, development, defense responses, etc. (Adie et al., 2007; Iqbal et al., 2017). Higher levels of *MYB44* expression in *C. papaya* compared to *N. benthamiana* indicate that the latter has managed to minimize the effect of the virion particles on the developmental stages of the plant. Another important TF is NAC042, which was shown to be a modulator of defense response in *N. benthamiana* during viral infections (Ke et al., 2022). It was upregulated in both host and non-host, but like *MYB44*, the expression was higher in the host plant, signifying infection severity. Another important TF is bZIP60, which is involved in ER stress response as it regulates the genes involved in unfolded protein response (Xu et al., 2019). Its significant upregulation in host plants designates that these plants are under severe stress due to the presence of virion particles. However, no significant change of *bZIP60* expression in the non-host plant may be pointing toward a better resistance mechanism against the virus. ERF5 is necessary for plant innate immunity (Son et al., 2012). Elevated viral load has resulted in the expression of *ERF5* being upregulated by 12-fold in the natural host, whereas just 1.2-fold increased expression in the non-host. This signifies that the non-host system has dealt with the adversity of the infection (Figures 4C,D).

Plant phenotypes can be significantly affected by viral infections, which can cause various symptoms that hinder growth, development, and productivity (Roossinck, 2010). The symptoms occur due to intricate molecular interactions between the virus and the host plant (Jiang and Zhou, 2023). Cytokinins (CKs) are plant hormones that stimulate cell growth and elongation and are instrumental in several developmental processes, such as transmitting nutritional signals and postponing senescence (Sakakibara, 2006). Additionally, CKs have been found to contribute to plant resistance against viral infections (Choi et al., 2011). Two essential genes involved in the cytokinin biosynthesis pathway are *IPT1* and *LOG1* (Takei et al., 2001; Kurakawa et al., 2007). The significantly decreased transcript expressions of these two genes in the host plant might suggest the hindrance in CK production due to virus assembly, which ultimately contributes to the distorted phenotype and wilted leaves in plants. Contrarily, unchanged expressions of these two genes in non-host plants point toward its unaffected CK biosynthesis, correlated with unaltered phenotype and non-chlorotic leaves. During pathogen invasion, Jasmonic acid (JA) acts as a vital phytohormone in defense mechanisms (Sun et al., 2011). In the absence of JA, symptoms of PVY-PVX co-infection develop at an accelerated rate, whereas treatment with JA enhances resistance against double infection (García-Marcos et al., 2013). The biosynthesis pathway of JA involves the *AOC1* and *OPR2* genes (Eng et al., 2021). Significant downregulation of these genes indicates the impeded JA biosynthesis and, consequently, compromised virus resistance. Nevertheless, despite the occurrence of viral replication, maintained JA biosynthesis as proved by unaltered expressions of said genes, justifying the futile impact of the virus on the physiology of non-host plants.

Autophagy delivers non-functional intracellular components to vacuoles to regulate the host’s response to pathogens like viruses (Shoji-Kawata and Levine, 2009). ATG8 proteins are crucial in forming autophagosomes and selecting cargo for degradation. (Nakatogawa et al., 2007; Johansen and Lamark, 2011). The expression of *ATG8f* is significantly suppressed during viral infection, indicating that the host is unable to undergo autophagy. However, maintained *ATG8f* expression in the non-host suggesting the occurrence of autophagy. Plants use PCD to defend themselves against different pathogens (Jorgensen et al., 2017). BI-1 is a well-known suppressor of PCD (Watanabe and Lam, 2009). The virus has likely overcome the host’s resistance through PCD, as shown by the significant increase in *BI-1* expression in the host. Conversely, while there is a change in BI-1 expression in the non-host, it is relatively less pronounced as compared to the host plant, suggesting effective usage of PCD.

**Figure 5:**
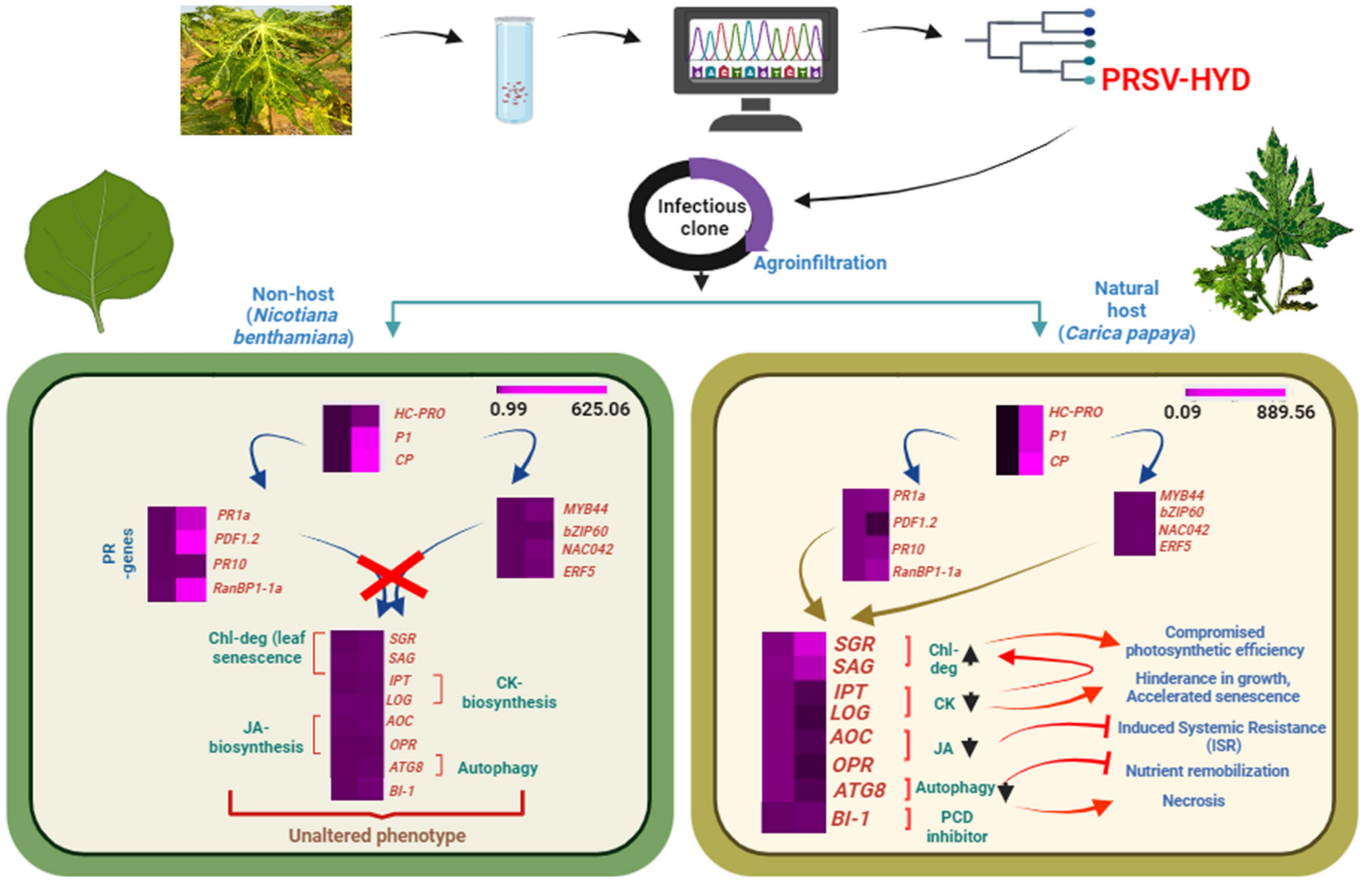
Schematic representation of the study. Depiction of complete genome sequencing and icDNA clone preparation of PRSV-HYD isolate, followed by agroinoculation in host (*Carica papaya*) and non-host (*Nicotiana benthamiana*) plants. Putative expression analysis of essential genes of both host and non-host plants showcasing the indispensability of host factors for effective systemic infection in host plant.

The accumulation of viral CP inside chloroplasts was related to the induction of chlorotic symptoms during *Tobacco mosaic virus* (TMV) infection (Lehto et al., 2003). It has been reported that *Sugarcane mosaic virus* (SCMV) HC-Pro has a specific interaction with the maize chloroplast precursor protein of Fd V, ultimately resulting in disturbances to the structure and function of chloroplasts (Cheng et al., 2008). Staygreen-1 (*Sgr-1*) regulates chlorophyll degradation during leaf senescence (Park et al., 2007). Based on current research, *SAG12* is considered the most effective molecular marker for identifying senescence (Weaver et al., 1998). The chlorotic spots on the symptomatic leaves of the host plants are justified by the upregulated *SGR1* and *SAG12* gene expressions. On the other hand, the absence of any chlorotic symptom or leaf senescence in the non-host could be due to the considerably lower expression levels of SGR1 and SAG12 compared to the host plant (Figures 4E,F). This entire study has been represented as a schematic diagram (Figure 5).

## 5 Conclusion

This study was conducted to characterize the PRSV-HYD isolate through complete genome sequencing. To our knowledge, it is the first report of PRSV isolate from South India to be fully sequenced. The PRSV-HYD genome differs significantly from other PRSV isolates in certain genomic regions, suggesting its evolving nature. It is essential to monitor the different circulating strains of PRSV to understand the phenotypic and genotypic characteristics of the infected host plants. Our comprehensive genome sequence data will aid future PRSV epidemiology and sequence diversity research. Moreover, this study is the first of its kind to report a complete infectious cDNA clone of PRSV from the Indian subcontinent. Despite the agroinfectivity of pCB301-icPRSV-HYD in both the host (*C. papaya*) and non-host (*N. benthamiana*) plants, only the host exhibited systemic infection with compromised expressions of genes related to cytokinin, jasmonic acid, and autophagy. Additionally, the virulence also affected the photosynthetic ability of the host by elevating chlorophyll degradation and consequently increasing chlorosis. Although the ability of this virus to be replicated, due to some unknown mechanism, the infectivity was subdued in non-host, proving the essential requirement of host factors for a successful systemic PRSV infection. This icDNA clone can be aided in the further detailed study of this virus at the molecular level, investigate virus-vector interactions, or use it as a viral vector to express heterologous proteins.

## Data availability statement

The datasets presented in this study can be found in online repositories. The names of the repository/repositories and accession number(s) can be found in the article/Supplementary material.

## Author contributions

PG: Conceptualization, Data curation, Formal analysis, Investigation, Methodology, Project administration, Software, Validation, Writing – original draft. PP: Conceptualization, Investigation, Methodology, Writing – original draft. LS: Conceptualization, Data curation, Investigation, Methodology, Writing – original draft, Software, Validation. HS: Investigation, Methodology, Writing – original draft. GP: Investigation, Validation, Writing – review & editing. KG: Conceptualization, Formal analysis, Funding acquisition, Investigation, Project administration, Resources, Supervision, Validation, Visualization, Writing – review & editing.

## Footnotes

^1^https://www.ncbi.nlm.nih.gov/orffinder/
