# Supplementary material for: Complete genome sequencing and construction of full-length infectious cDNA clone of papaya ringspot virus-HYD isolate and its efficient in planta expression
Source: Front Microbiol. 2023 Nov 30;14:1310236. doi: 10.3389/fmicb.2023.1310236 (PMC10721977; doi:10.3389/fmicb.2023.1310236)
Supplement: Supplementary file 1 [file Data_Sheet_1.docx]

**Supplementary Table S1.** List of primers used for complete genome sequencing and infectious clone preparation

| **SL. No** | **Name of the Primer** | **Sequence (5′-3′)** |
| --- | --- | --- |
| 1 | Start 1-29+ | AAATAAAACATCTCAACACAACAC |
| 2 | 551+ | GCTGACGCGGTTGATTTTGC |
| 3 | 551- | GCAAAATCAACCGCGTCAGC |
| 4 | 1535+ | GGACACAAACCGCGAAAATCTGC |
| 5 | 1554- | GATTTTCGCGGTTTGTGTCC |
| 6 | 1764- | GCCCTATTGAAACCGAGC |
| 7 | 2198+ | CGAGAGATTGTGAGATGGC |
| 8 | 3335- | CCTTCCTGCACAATTGC |
| 9 | 3040+ | GCTAATCCAATTTGCTAGG |
| 10 | 3335- | CCTTCCTGCACAATTGCTCC |
| 11 | 3558- | GTGCGGTGTCAGATCGACTTCCAATGC |
| 12 | 3700+ | CTTCTCACCGAAGTATTGTGTACAC |
| 13 | 3768- | CCAGCTCCAGCTTTAACCGG |
| 14 | 4610+ | GAGCCCACCAGACCTCTTTGTGAG |
| 15 | 5028- | GCAAAGGCAGTCACATCACTATTCG |
| 16 | 5238- | GCTTCTATGTCAAGTGTGACG |
| 17 | 6200+ | GGAATCAAGCATATAAAGCGAGAGC |
| 18 | 6790- | GCATTGGAAGTCGATCTGACACCGCAT |
| 19 | 6858+ | GACAAACAGGCCCGCCTAG |
| 20 | 6900- | GGAACCTGGTCAACTTGAATAGTCC |
| 21 | 7102- | GGATTTCACTATGAGTTCACC |
| 22 | 7688+ | GTAGGTTCCGCTCCTAGCAATTTG |
| 23 | 8088- | GCTTGTCTATGTTCCACTGTCGAACC |
| 25 | 8291+ | CCATGGACCGTCGGAATG |
| 26 | 8488- | CTTAAGCATTTGCTCTCCAATATCC |
| 27 | 8982+ | CACCAGATTCGCAGATTTTACCAATG |
| 28 | 9129- | CTAATTCATCCATCGAACCACGTTC |
| 29 | 9154+ | GAGCGTGGAGATTCTCCTG |
| 30 | 9311- | CCTTTTCTTTCTCCTTCAGC |
| 31 | 9652+ | GGTTTAATGGTTTGGTGTATCG |
| 32 | 10,317- | GCTCATTCTAAGAGGCTCG |
| 33 | 5′ RACE-I | CCTTCACTTATAACAAACTCACC |
| 34 | 5′ RACE- II | GCTCGATCCATCCGCTTCC |
| 35 | 3′ RACE | GGTGAATTCAAAAACACC |
| 36 | Oligo (dT) | TTTTTTTTTTTTTTT |
| 37 | 35S fusion 1 F | AGATCTCCTGTGGTTGGC |
| 38 | 35S fusion 1 R | GTGTTGAGATGTTTTATTTCTCTCCAAATGAAATGAACTTCC |
| 39 | 35S fusion 2 F | GGAAGTTCATTTCATTTGGAGAGAAATAAAACATCTCAACACAACAC |
| 40 | 35S fusion 2 R | GGCTTATTGTTTCGATCCATCAGC |
| 41 | RZ fusion 1 F | GGTGTTAAGACGAGAAAGTTCG |
| 42 | RZ fusion 1 R | GTGAATCATGTCTTGATTTTTTTTTTTTTTTTTTTTTTTGCTCATTCTAAGAGGCTCGAATAACACGTGGG |
| 43 | RZ fusion 2 R | GCCCGGGCCGTTTCGTCCTCACGGACTCATCAGAAGACATGTGAATCATGTC |
| 44 | P1 F | CCATGTCTTCGTTATATCAATTGC |
| 45 | P1 R | CGTATTGTTCCATTTTAGC |
| 46 | HC-Pro F | CCAATGACGTGGCTGAAAAATTCC |
| 47 | HC-Pro R | ACCAACAATGTAGTGCTTC |
| 48 | CP F | TCCAAGACTGAAGCAGTGG |
| 49 | CP R | TCAGTTGCGCATACCCAGG |

**Supplementary Table S2**. List of primers used for real-time PCR analysis

| **SL No.** | **Primer name** | **Primer sequence (5′-3′)** |
| --- | --- | --- |
| 1 | PRSV P1 F | GAGGAAAGGTGGAAGCTATG |
| 2 | PRSV P1 R | GCGGTTTGTGTCCAACTA |
| 3 | PRSV HC-Pro F | CGGCTCTCGCAACAATAA |
| 4 | PRSV HC-Pro R | TCAACTCCTCACCGACTT |
| 5 | PRSV CP F | GTCATGATGGATGGTGAGAC |
| 6 | PRSV CP R | CTGCCGCGTTACTGAAAT |
| 7 | NB PR1a F | GATGTGGGTCGATGAGAAAC |
| 8 | NB PR1a R | GCACATCCAACACGAACT |
| 9 | NB PDF 1.2 F | GACCATGCTCAAGAGATAGC |
| 10 | NB PDF 1.2 R | GCAAGGCCTAGTACAGAAAC |
| 11 | NB PR10 F | GAGGTCACAACATCAGCTTC |
| 12 | NB PR10 R | CCATCACCCTCAACAATCTC |
| 13 | NB RanBP1-1a F | GAGAGCCTGAATTGGAGAAG |
| 14 | NB RanBP1-1a R | TCACCAGTGGAGACAGAA |
| 15 | NB MYB44 F | CAGCATCGACAGAGTTAAGG |
| 16 | NB MYB44 R | TTCAAACTGGCTGGGTTC |
| 17 | NB BI F | CCAGATCTCTCCCTTTGTTC |
| 18 | NB BI R | TCCCAGCGTCGTAAGTAA |
| 19 | NB bZIP60 F | CGCAAGAGGCAATTGAGA |
| 20 | NB bZIP60 R | GACAGCACTGGAGAACTAAC |
| 21 | NB NAC042 F | AACTGGCTGCTACCCTTA |
| 22 | NB NAC042 R | GCTACTCCCTTCCTTTGAAC |
| 23 | NB ERF5 F | GCTTCGAGTTTGGGACAA |
| 24 | NB ERF5 R | CTCCTGCTTCACTGGTATTG |
| 25 | NB SGR1 F | GCAACTGGGATTTACCACAAC |
| 26 | NB SGR1 R | CTCACCATCAGTCCCAGAAATA |
| 27 | NB SAG12 F | CTACGGAAGGGCTACACCAAC |
| 28 | NB SAG12 R | GGCAGTGTCCAAGAGTCCACC |
| 29 | NB ATG 8f F | ACAGTAGGGCAGTTTGTCTATG |
| 30 | NB ATG 8f R | CCTGTTGGTGGTAGGACATTAT |
| 31 | NB IPT1 F | GAAGAAGCAGCGAAGGAGATAA |
| 32 | NB IPT1 R | CGTGGCATCTACTCTTTGTAGG |
| 33 | NB LOG1 F | TGGAGGAGGAAGTATTGGTTTG |
| 34 | NB LOG1 R | GGCATGAGAGTCCTTGGAATTA |
| 35 | NB AOC3 F | GTGGTAGCCCTGCTTATCTTC |
| 36 | NB AOC3 R | TGCAGAGTCCTGCTGTTATTC |
| 37 | NB OPR2 F | GTAGGGAGAGTTTCCCACAAAG |
| 38 | NB OPR2 R | GCACGAATTTGAGGTGCTAATG |
| 39 | CP MYB44-like F | GTCCATCAGGCTCCGATTT |
| 40 | CP MYB44-like R | CGATATGCTGAGTTGGAGGTAG |
| 41 | CP BI-1 F | TCTGGTGTGTCCATGCTTATG |
| 42 | CP BI-1 R | GGGTGTCCACCACAATATATCC |
| 43 | CP bZIP60-Like F | CTCCGCTTGTAGTGAGGATTT |
| 44 | CP bZIP60-Like R | GTAGCCGTCTTCGTTGATGT |
| 45 | CP NAC089-like F | CGACACCGAGTTGATCTCTTAC |
| 46 | CP NAC089-like R | AGTCCCAGGGCTCGTATTTA |
| 47 | CP ERF5-like F | AGTTTCGAGCTCTGCCTATTC |
| 48 | CP ERF5-like R | GCTTCCACTTTCAGCAAGATTT |
| 49 | CP SGR1 F | CTCTCACAAGAACGCACATTTC |
| 50 | CP SGR1 R | CCTTACGGCCTCAGAAACAA |
| 51 | CP SAG39 F | GCCTCACAACTGAGACCAATTA |
| 52 | CP SAG39 R | GTTGGCTGGTACGTCTTCATAG |
| 53 | CP ATG 8f F | CAACGTGCTTCCACCTACA |
| 54 | CP ATG 8f R | CGTGACATAGAGAAACCCATCTT |
| 55 | CP IPT1 F | TCATCTGGGTCGACGTATCA |
| 56 | CP IPT1 R | CATAGAACTCGGCCAACTCTTC |
| 57 | CP LOG1 F | GGTTGATGGGTCTGGTTTCT |
| 58 | CP LOG1 R | CTACTGCATGGCCTGTTATCT |
| 59 | CP AOC3 F | CTGCAGAAACGCATTGGAATAA |
| 60 | CP AOC3 R | ACCGTAGTCTCCGAAGTAGAA |
| 61 | CP OPR2 F | AGCAGACAGGGTTGGAATTAG |
| 62 | CP OPR2 R | AGGACTCAGCCATGTAGAGA |
