# Supplementary material for: Complete genome sequencing and construction of full-length infectious cDNA clone of papaya ringspot virus-HYD isolate and its efficient in planta expression
Source: Front Microbiol. 2023 Nov 30;14:1310236. doi: 10.3389/fmicb.2023.1310236 (PMC10721977; doi:10.3389/fmicb.2023.1310236)
Supplement: Supplementary file 2 [file Presentation_1.pptx]

## Slide 1
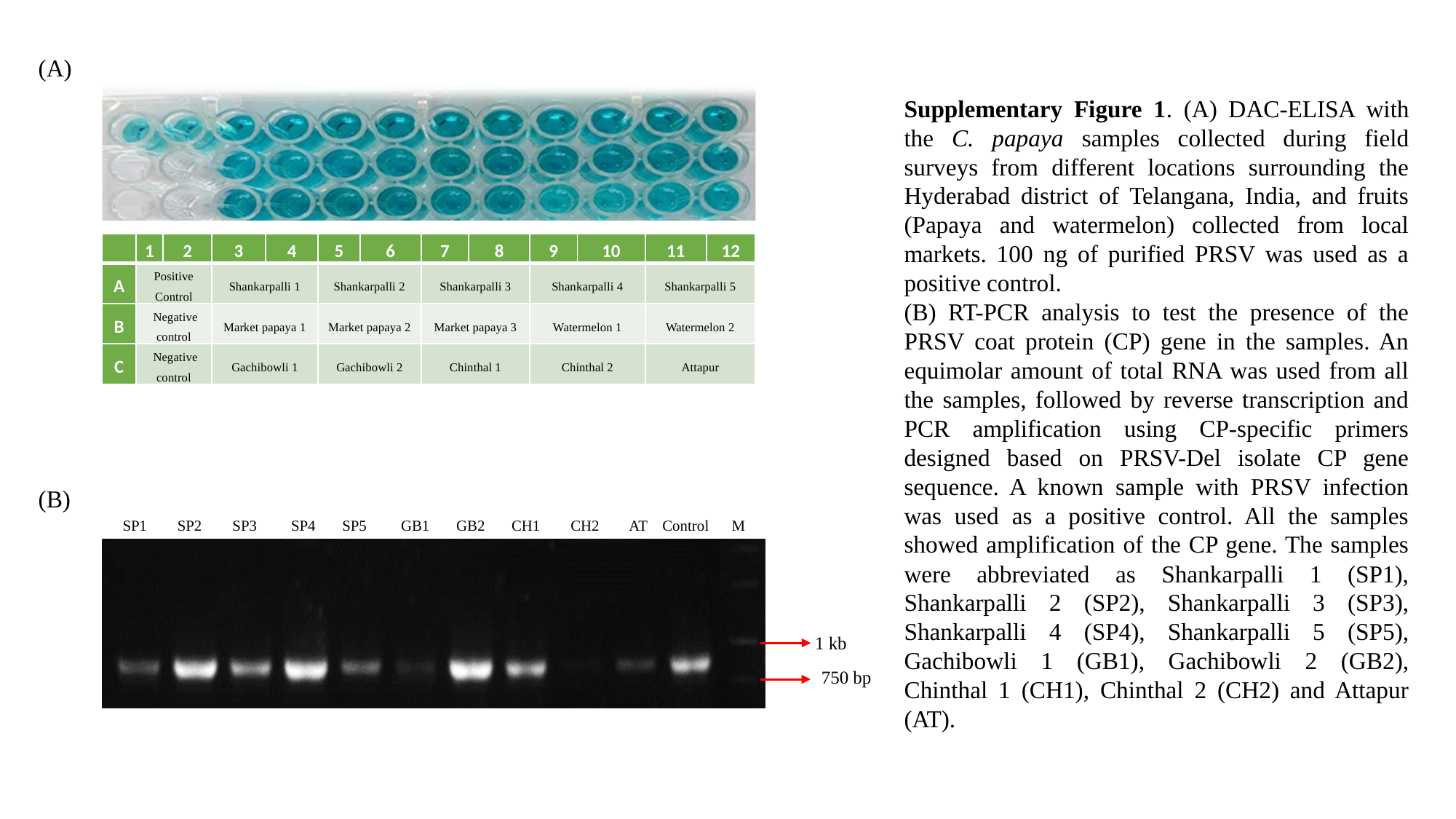

(A)
Supplementary Figure 1. (A) DAC-ELISA with the C. papaya samples collected during field surveys from different locations surrounding the Hyderabad district of Telangana, India, and fruits (Papaya and watermelon) collected from local markets. 100 ng of purified PRSV was used as a positive control.
(B) RT-PCR analysis to test the presence of the PRSV coat protein (CP) gene in the samples. An equimolar amount of total RNA was used from all the samples, followed by reverse transcription and PCR amplification using CP-specific primers designed based on PRSV-Del isolate CP gene sequence. A known sample with PRSV infection was used as a positive control. All the samples showed amplification of the CP gene. The samples were abbreviated as Shankarpalli 1 (SP1), Shankarpalli 2 (SP2), Shankarpalli 3 (SP3), Shankarpalli 4 (SP4), Shankarpalli 5 (SP5), Gachibowli 1 (GB1), Gachibowli 2 (GB2), Chinthal 1 (CH1), Chinthal 2 (CH2) and Attapur (AT).
| | 1 | 2 | 3 | 4 | 5 | 6 | 7 | 8 | 9 | 10 | 11 | 12 |
| --- | --- | --- | --- | --- | --- | --- | --- | --- | --- | --- | --- | --- |
| A | Positive Control | | Shankarpalli 1 | | Shankarpalli 2 | | Shankarpalli 3 | | Shankarpalli 4 | | Shankarpalli 5 | |
| B | Negative control | | Market papaya 1 | | Market papaya 2 | | Market papaya 3 | | Watermelon 1 | | Watermelon 2 | |
| C | Negative control | | Gachibowli 1 | | Gachibowli 2 | | Chinthal 1 | | Chinthal 2 | | Attapur | |
(B)
SP1 SP2 SP3 SP4 SP5 GB1 GB2 CH1 CH2 AT Control M
1 kb
750 bp

## Slide 2
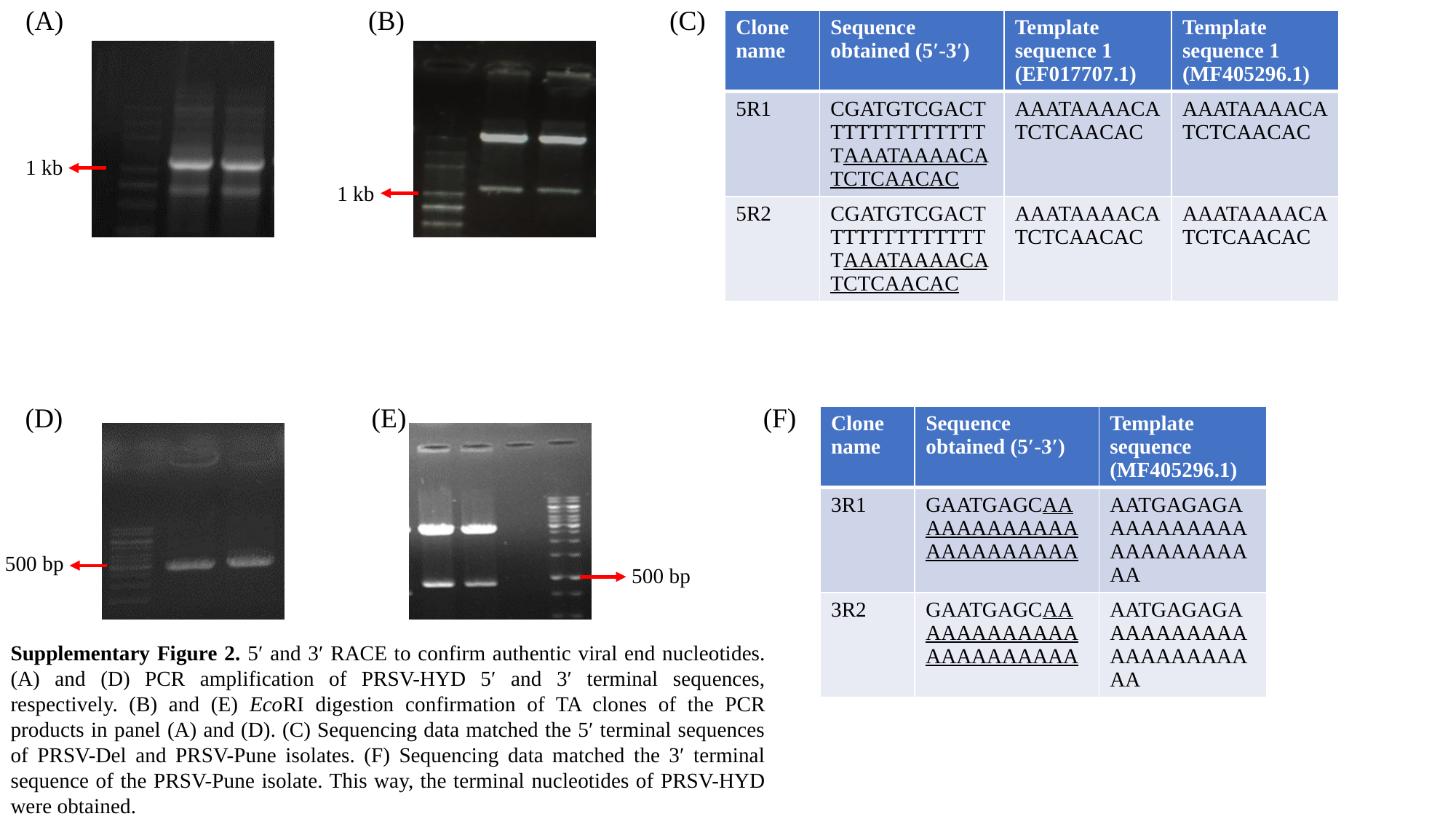

(A)
(B)
(C)
| Clone name | Sequence obtained (5′-3′) | Template sequence 1 (EF017707.1) | Template sequence 1 (MF405296.1) |
| --- | --- | --- | --- |
| 5R1 | CGATGTCGACTTTTTTTTTTTTTTAAATAAAACATCTCAACAC | AAATAAAACATCTCAACAC | AAATAAAACATCTCAACAC |
| 5R2 | CGATGTCGACTTTTTTTTTTTTTTAAATAAAACATCTCAACAC | AAATAAAACATCTCAACAC | AAATAAAACATCTCAACAC |
1 kb
1 kb
(D)
(E)
(F)
| Clone name | Sequence obtained (5′-3′) | Template sequence (MF405296.1) |
| --- | --- | --- |
| 3R1 | GAATGAGCAAAAAAAAAAAAAAAAAAAAAA | AATGAGAGAAAAAAAAAAAAAAAAAAAAA |
| 3R2 | GAATGAGCAAAAAAAAAAAAAAAAAAAAAA | AATGAGAGAAAAAAAAAAAAAAAAAAAAA |
500 bp
500 bp
Supplementary Figure 2. 5′ and 3′ RACE to confirm authentic viral end nucleotides. (A) and (D) PCR amplification of PRSV-HYD 5′ and 3′ terminal sequences, respectively. (B) and (E) EcoRI digestion confirmation of TA clones of the PCR products in panel (A) and (D). (C) Sequencing data matched the 5′ terminal sequences of PRSV-Del and PRSV-Pune isolates. (F) Sequencing data matched the 3′ terminal sequence of the PRSV-Pune isolate. This way, the terminal nucleotides of PRSV-HYD were obtained.

## Slide 3
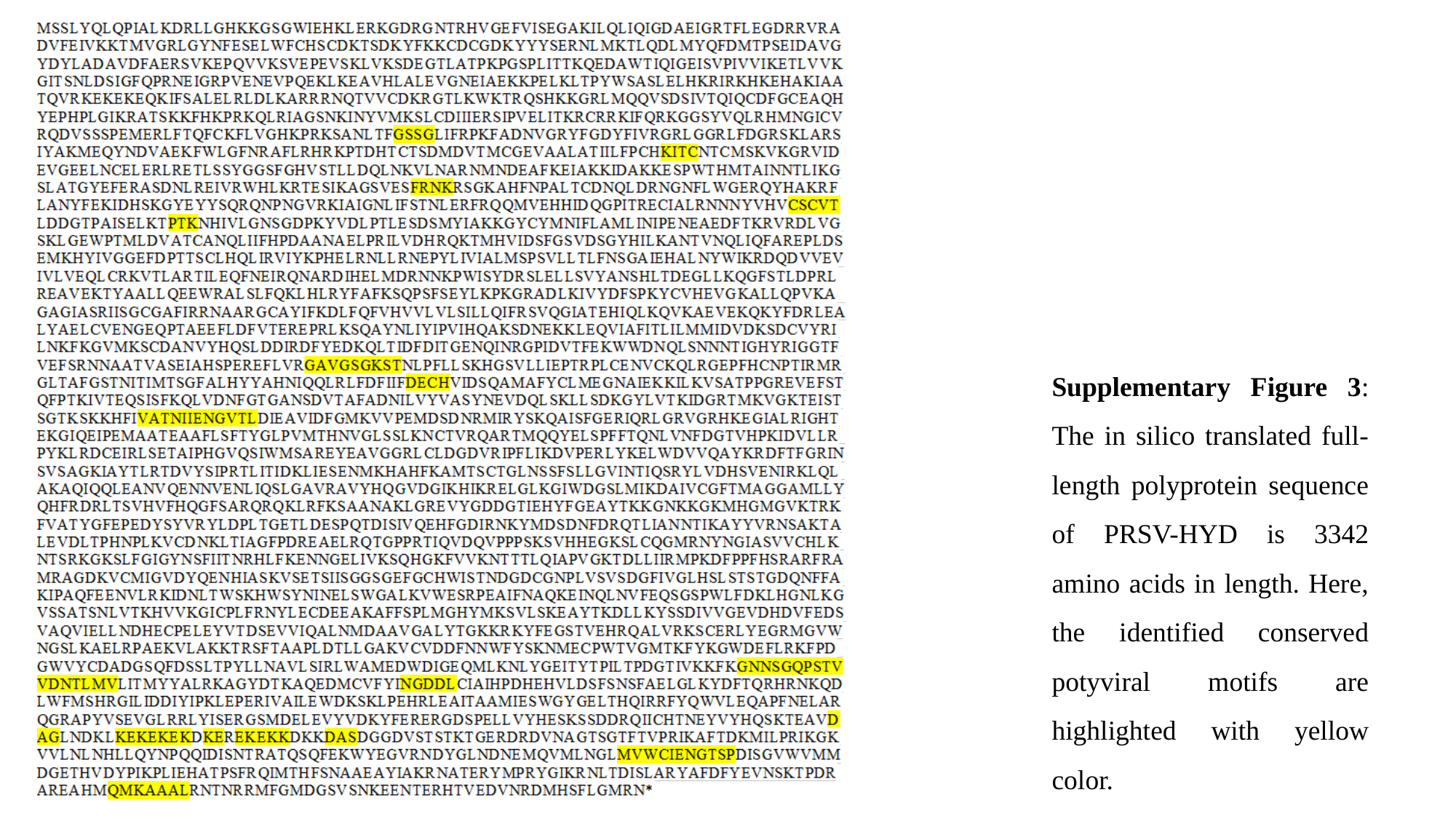

Supplementary Figure 3: The in silico translated full-length polyprotein sequence of PRSV-HYD is 3342 amino acids in length. Here, the identified conserved potyviral motifs are highlighted with yellow color.

## Slide 4
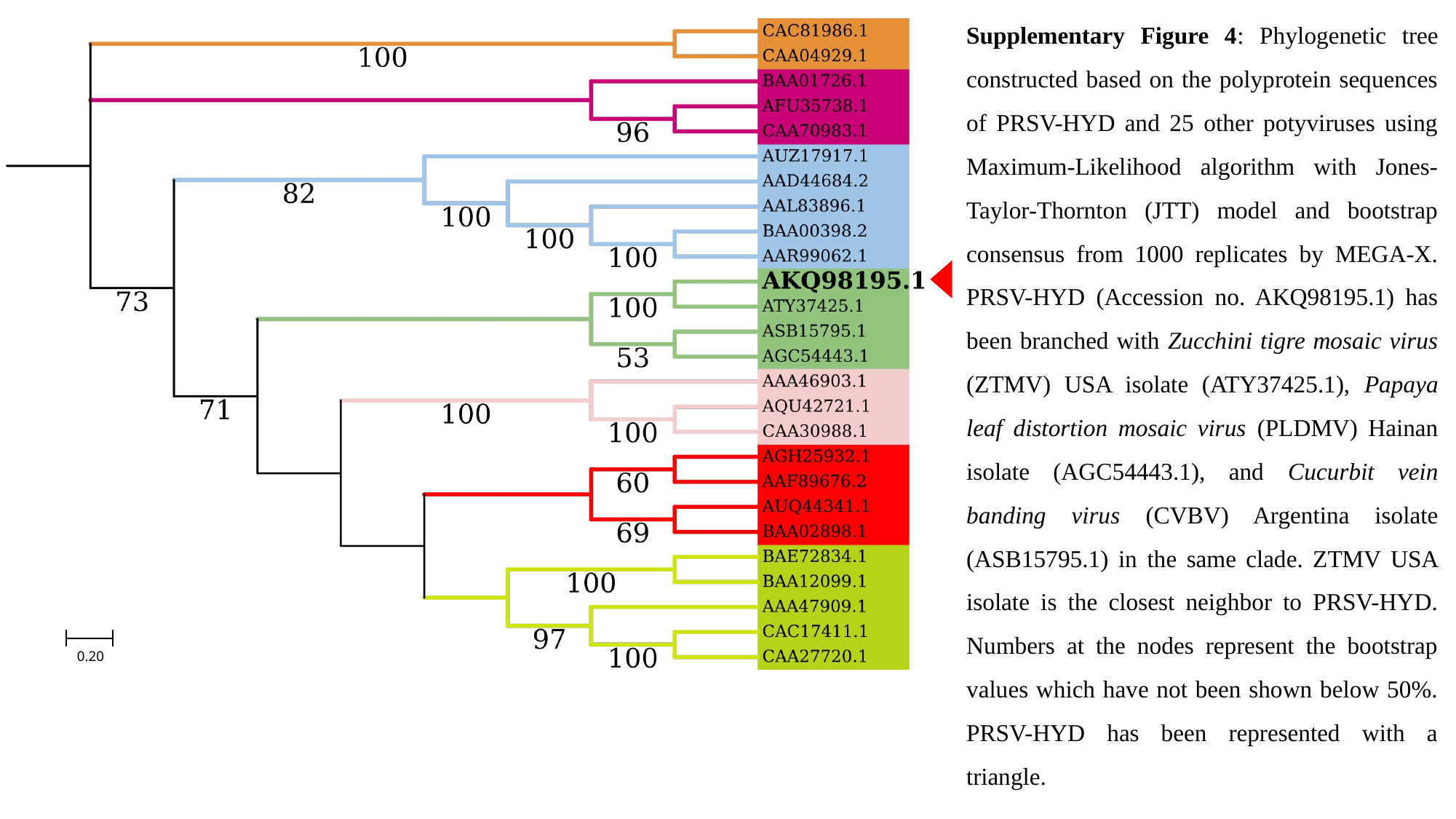

Supplementary Figure 4: Phylogenetic tree constructed based on the polyprotein sequences of PRSV-HYD and 25 other potyviruses using Maximum-Likelihood algorithm with Jones-Taylor-Thornton (JTT) model and bootstrap consensus from 1000 replicates by MEGA-X. PRSV-HYD (Accession no. AKQ98195.1) has been branched with Zucchini tigre mosaic virus (ZTMV) USA isolate (ATY37425.1), Papaya leaf distortion mosaic virus (PLDMV) Hainan isolate (AGC54443.1), and Cucurbit vein banding virus (CVBV) Argentina isolate (ASB15795.1) in the same clade. ZTMV USA isolate is the closest neighbor to PRSV-HYD. Numbers at the nodes represent the bootstrap values which have not been shown below 50%. PRSV-HYD has been represented with a triangle.
0.20

## Slide 5
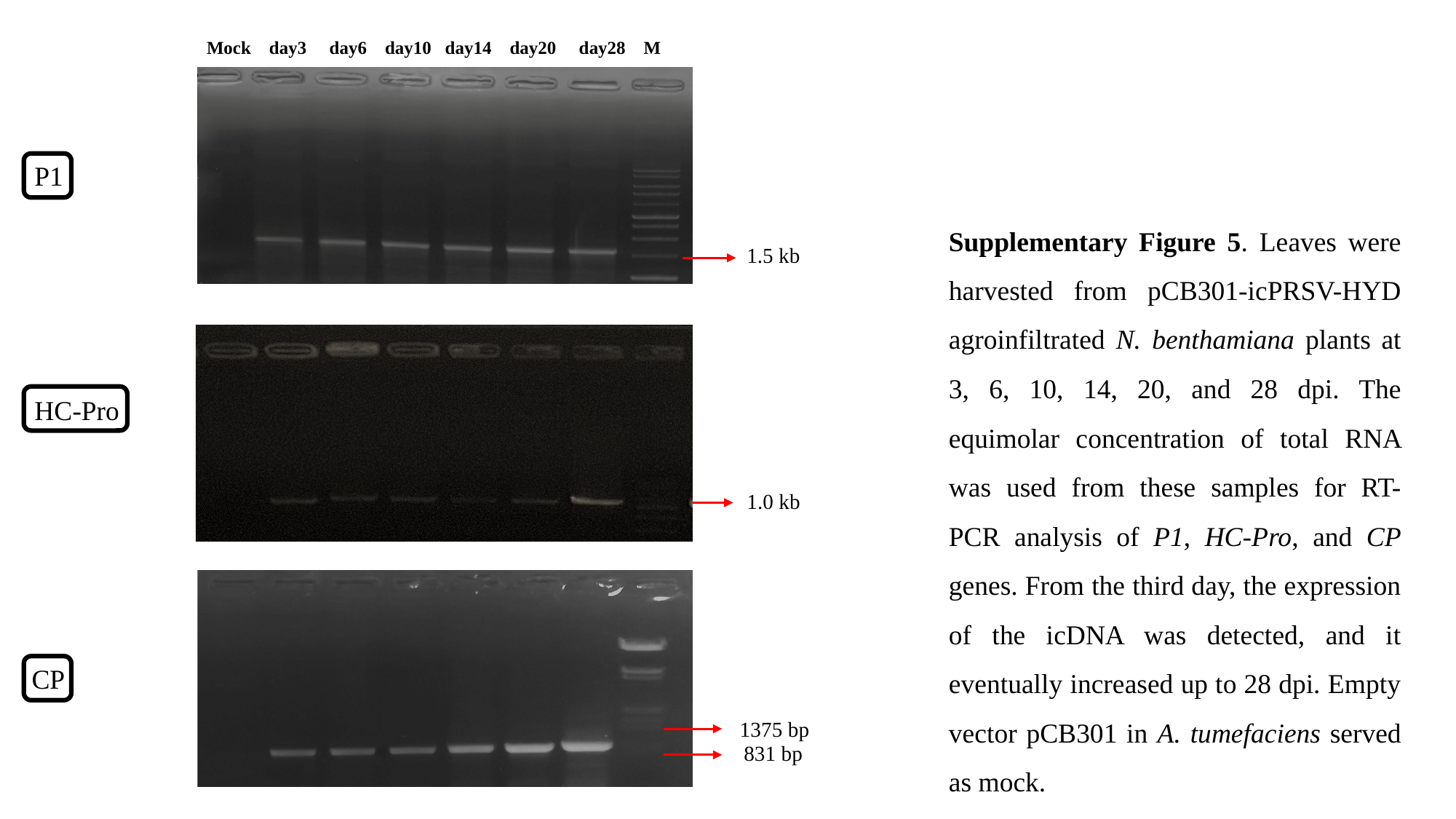

Mock day3 day6 day10 day14 day20 day28 M
P1
Supplementary Figure 5. Leaves were harvested from pCB301-icPRSV-HYD agroinfiltrated N. benthamiana plants at 3, 6, 10, 14, 20, and 28 dpi. The equimolar concentration of total RNA was used from these samples for RT-PCR analysis of P1, HC-Pro, and CP genes. From the third day, the expression of the icDNA was detected, and it eventually increased up to 28 dpi. Empty vector pCB301 in A. tumefaciens served as mock.
1.5 kb
HC-Pro
1.0 kb
CP
1375 bp
831 bp

## Slide 6
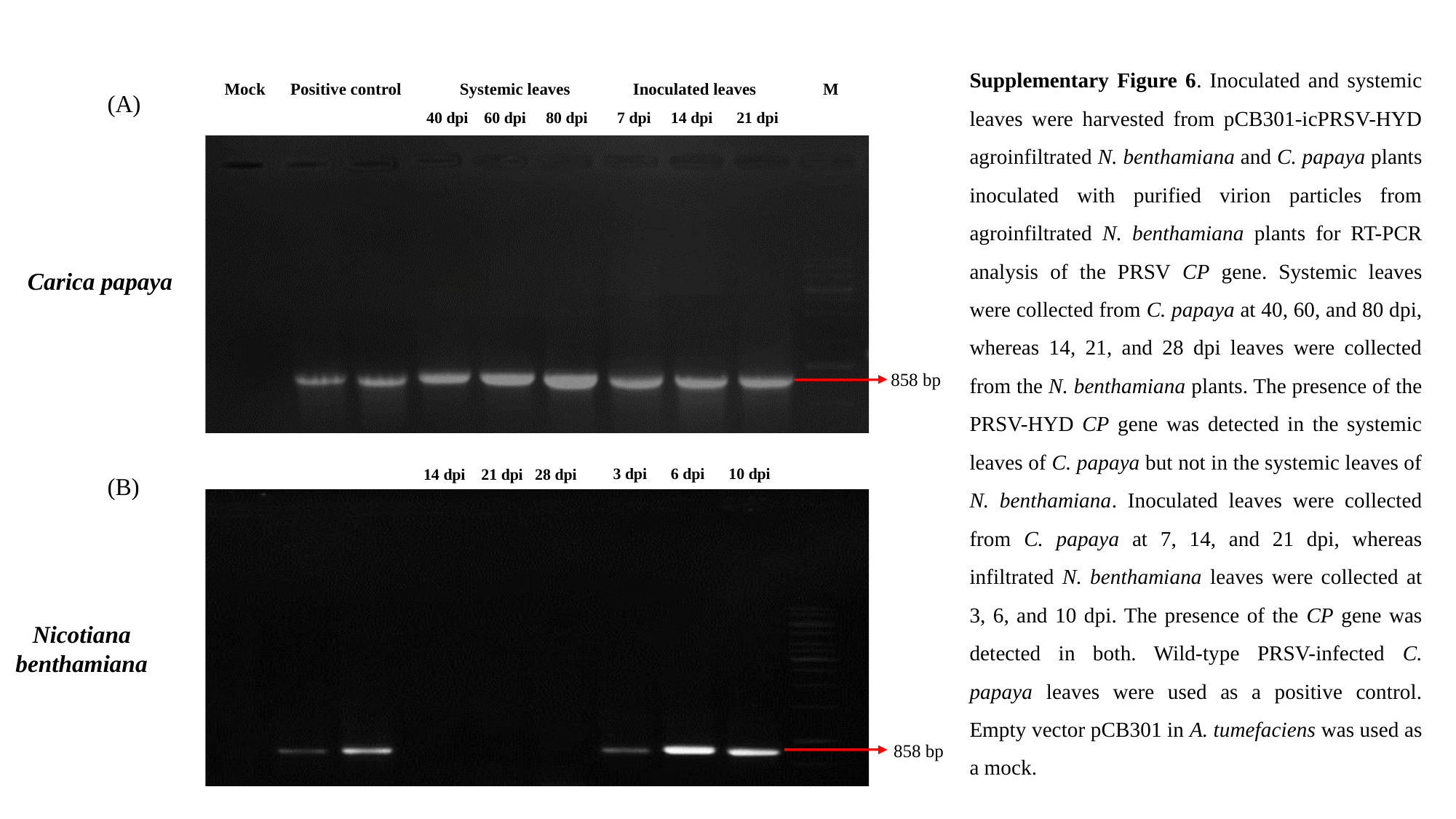

Supplementary Figure 6. Inoculated and systemic leaves were harvested from pCB301-icPRSV-HYD agroinfiltrated N. benthamiana and C. papaya plants inoculated with purified virion particles from agroinfiltrated N. benthamiana plants for RT-PCR analysis of the PRSV CP gene. Systemic leaves were collected from C. papaya at 40, 60, and 80 dpi, whereas 14, 21, and 28 dpi leaves were collected from the N. benthamiana plants. The presence of the PRSV-HYD CP gene was detected in the systemic leaves of C. papaya but not in the systemic leaves of N. benthamiana. Inoculated leaves were collected from C. papaya at 7, 14, and 21 dpi, whereas infiltrated N. benthamiana leaves were collected at 3, 6, and 10 dpi. The presence of the CP gene was detected in both. Wild-type PRSV-infected C. papaya leaves were used as a positive control. Empty vector pCB301 in A. tumefaciens was used as a mock.
Mock Positive control Systemic leaves Inoculated leaves M
(A)
40 dpi 60 dpi 80 dpi
 7 dpi 14 dpi 21 dpi
Carica papaya
858 bp
3 dpi 6 dpi 10 dpi
14 dpi 21 dpi 28 dpi
(B)
Nicotiana benthamiana
858 bp
